# Global Respiratory Syncytial Virus–Related Infant Community Deaths

**DOI:** 10.1093/cid/ciab528

**Published:** 2021-09-02

**Authors:** Natalie I Mazur, Yvette N Löwensteyn, Joukje E Willemsen, Christopher J Gill, Leah Forman, Lawrence M Mwananyanda, Dianna M Blau, Robert F Breiman, Shabir A Madhi, Sana Mahtab, Emily S Gurley, Shams El Arifeen, Nega Assefa, J Anthony G Scott, Dickens Onyango, Beth A Tippet Barr, Karen L Kotloff, Samba O Sow, Inacio Mandomando, Ikechukwu Ogbuanu, Amara Jambai, Quique Bassat, CHAMPS Network the RSV GOLD Study Group, Somsak Thamthitiwat, Angela Gentile, Maria Florencia Lucion, Márcia Rosane Pires, Fernanda de-Paris, Aubree Gordon, José Félix Sánchez, Marilla G Lucero, Socorro P Lupisan, Bradford D Gessner, Haoua Tall, Natasha Halasa, Najwa Khuri-Bulos, D James Nokes, Patrick K Munywoki, Grieven P Otieno, Katherine L O’Brien, Katherine L Oshitani, Maria Tereza da Costa Oliveira, Carla Cecília de Freitas Lázaro Emediato, Asad Ali, Uzma Bashir Aamir, Daniel E Noyola, Cheryl Cohen, Jocelyn Moyes, Heloisa Ihle Garcia Giamberardino, Jane Melissa Webler, Patricia Gomes de Matos Bezerra, Maria do Carmo Menezes Bezerra Duarte, Helen Y Chu, Rashmi Ranjan Das, Martin W Weber, Nusrat Homaira, Adam Jaffe, Katharine M Sturm-Ramirez, Wei Su, Chiang Chun Yuan, Sandra Chaves, Gideon O Emukule, Sergio de Andrade Nishioka, Felipe Cotrim de Carvalho, Şule Gökçe, Sonia M Raboni, Michael Hawkes, Melina Messaoudi, Juliet Bryant, Ghassan S Dbaibo, Rima Hanna-Wakim, J A A Sampath Jayaweera, Kirill Stolyarov, Piyarat Suntarattiwong, Tufária Mussá, Alfredo Bruno, Domenica de Mora, Nasamon Wanlapakorn, Zheng de Xie, Junhong Ai, Jenny Ojeda, Lida Zamora, Evangeline Obodai, John Kofi Odoom, Maha Talaat Ismail, Andrea Buchwald, Cristina O’Callaghan-Gordo, Jaime Fernandez-Sarmiento, Evelyn Obando-Belalcazar, Tapan Dhole, Sheetal Verma, Aykut Eşki, G Ozturk Kartal, Mohammed Al Amad, Abdul Wahed Al Serouri, Yoke FunChan, Jamal I-Ching Sam, Daniel Jarovsky, Daniella Gregória Bomfim Prado da Silva, José Gareca Perales, Teck-Hock Toh, Jeffrey Lee Soon Yit, Tanil Kendirli, Emrah Gun, Tani Sagna, Serge Diagbouga, Fahmida Chowdhury, Md Ariful Islam, Marietjie Venter, Adele Visser, Minh-Hong Pham, Pablo Vásquez-Hoyos, Sebastián González-Dambrauskas, Franco Díaz Rubio, Todd Karsies, Eliana Zemanate, Ledys Izquierdo, Rubén Lasso Palomino, Rosalba Pardo-Carrero, Reginna Grigolli-Cesar, Soledad Menta, Nicolás Monteverde, Muhterem Duyu, Senjuti Saha, Samir K Saha, Matthew Kelly, Marcela Echavarria, Tuan Tran, Aida Borgi, Ahmed Ayari, Mauricio T Caballero, Fernando P Polack, Saad Omer, Abdul Momin Kazi, Eric A F Simões, Ashish Satav, Louis J Bont

**Affiliations:** 1 Division of Infectious Diseases, Department of Pediatrics, University Medical Centre Utrecht, Utrecht, The Netherlands; 2 Department of Global Health, Boston University School of Public Health, Boston, Massachusetts, USA; 3 Centers for Disease Control and Prevention, Atlanta, Georgia, USA; 4 Emory University, Global Health Institute, Child Health and Mortality Prevention Surveillance (CHAMPS) Network, Atlanta, Georgia, USA; 5 South African Medical Research Council—Vaccines and Infectious Diseases Analytics Research Unit, School of Pathology, Faculty of Health Sciences, University of the Witwatersrand, Johannesburg, South Africa; 6 Department of Science and Technology/National Research Foundation—Vaccine Preventable Diseases Unit, University of the Witwatersrand, Johannesburg, South Africa; 7 Johns Hopkins Bloomberg School of Public Health, Johns Hopkins University, Baltimore, Maryland, USA; 8 International Centre for Diarrheal Disease Research, Bangladesh (icddr,b), Dhaka, Bangladesh; 9 College of Health and Medical Sciences, Haramaya University, Harar, Ethiopia; 10 London School of Hygiene and Tropical Medicine, London, United Kingdom; 11 Kisumu County Department of Health, Kisumu, Kenya; 12 Centers for Disease Control, Kisumu, Kenya; 13 Department of Pediatrics, Center for Vaccine Development and Global Health and Division of Infectious Disease and Tropical Pediatrics, University of Maryland School of Medicine, Baltimore, Maryland, USA; 14 Center for Vaccine Development, Bamako, Mali; 15 Centro de Investigação em Saúde de Manhiça (CISM), Maputo, Mozambique; 16 Instituto Nacional de Saúde (INS), Ministério da Saúde, Maputo, Mozambique; 17 Crown Agents Sierra Leone, Freetown, Sierra Leone; 18 Ministry of Health and Sanitation, Freetown, Sierra Leone; 19 ISGlobal, Hospital Clínic–Universitat de Barcelona, Barcelona, Spain; 20 Catalan Institution for Research and Advanced Studies (ICREA), Pg. Lluís Companys 23, Barcelona, Spain; 21 Department of Pediatrics, Hospital Sant Joan de Déu, Universitat de Barcelona, Esplugues, Barcelona, Spain; 22 Consorcio de Investigación Biomédica en Red de Epidemiología y Salud Pública (CIBERESP), Madrid, Spain; 23 Fundación Infant, Buenos Aires, Argentina; 24 Consejo Nacional de Investigaciones Científicas y Técnicas (CONICET), Buenos Aires, Argentina; 25 Epidemiology of Microbial Diseases, Yale School of Public Health, Yale University, New Haven, Connecticut, USA; 26 Yale Institute for Global Health, New Haven, Connecticut, USA; 27 The Aga Khan University, Karachi, Pakistan; 28 Department of Pediatrics and Center for Global Health, University of Colorado, Children’s Hospital Colorado, Aurora, Colorado, USA; 29 Mahatma Gandhi Tribal Hospital, Kadhava, Maharashtra, India; 30 Respiratory Syncytial Virus Network (ReSViNET) Foundation, Zeist, The Netherlands; 1001 Division of Global Health Protection, Thailand Ministry of Public Health—US Centers for Disease Control and Prevention Collaboration, Nonthaburi, Thailand; 1002 Department of Epidemiology, Ricardo Gutiérrez Children Hospital, Buenos Aires, Argentina; 1003 Hospital de Clínicas de Porto Alegre, Universidade Federal do Rio Grande do Sul, Porto Alegre, Brazil; 1004 Department of Epidemiology, School of Public Health, University of Michigan, Michigan, USA; 1005 Department of Medicine, Hospital de Referencia Manuel de Jesús, Managua, Nicaragua; 1006 Research Institute for Tropical Medicine, Muntinlupa City, Philippines; 1007 Agence de Médecine Préventive, Paris, France; 1008 Vanderbilt University Medical Center, Nashville, Tennessee, USA; 1009 Department of Pediatrics, University of Jordan, Aljubeiha, Amman, Jordan; 1010 Kenya Medical Research Institute, Wellcome Trust Research Programme, Centre for Geographic Medicine Research—Coast, Kilifi, Kenya; 1011 International Vaccine Access Center, Pneumonia Etiology Research for Child Health (PERCH), Johns Hopkins Bloomberg School of Public Health, Baltimore, Maryland, USA; 1012 Department of Virology, Tohoku University Graduate School of Medicine, Aoba-ku, Sendai, Miyagi, Japan; 1013 Health Secretariat of the City of Belo Horizonte, Belo Horizonte, Brazil; 1014 Department of Pediatrics and Child Health, Aga Khan University, Karachi, Pakistan; 1015 Department of Virology, National Institute of Health, Islamabad, Pakistan; 1016 Faculty of Medicine, Department of Microbiology, Universidad Autónoma de San Luis Potosí, San Luis Potosí, Mexico; 1017 Centre for Respiratory Disease and Meningitis, National Institute for Communicable Diseases, Johannesburg, South Africa; 1018 Hospital Pequeno Príncipe, Curitiba, Brazil; 1019 Instituto de Medicina Integral Prof. Fernando Figueira (IMIP), Recife, Brazil; 1020 Division of Allergy & Infectious Diseases, Department of Medicine, University of Washington, Seattle, Washington, USA; 1021 All India Institute of Medical Sciences (AIIMS), Bhubaneswar, India; 1022 Department of Child and Adolescent Health and Development, World Health Organization, Geneva, Switzerland; 1023 Medical Research Council Laboratories, Fajara, The Gambia; 1024 School of Women’s and Children’s Health, Faculty of Medicine, University of New South Wales, Sydney, New South Wales, Australia; 1027 Can Am International Medical Center, Guangzhou, China; 1028 National Center for Immunization and Respiratory Diseases, Centers for Disease Control and Prevention, Nairobi, Kenya; 1029 Department of Transmissible Diseases, Ministry of Health, Brasília, Brazil; 1030 Instituto Oswaldo Cruz-Fiocruz, Rio de Janeiro, Brazil; 1031 Department of Pediatrics, General Pediatrics Unit, Ege University, Izmir, Turkey; 1032 Virology Laboratory, Department of Infectious Diseases, Hospital de Clínicas, Federal University of Paraná, Curitiba, Brazil; 1033 Department of Pediatrics, Stollery Children’s Hospital, University of Alberta, Edmonton, Alberta, Canada; 1034 Emerging Pathogens Laboratory—Fondation Mérieux, Centre International de Recherche en Infectiologie, Lyon, France; 1035 Department of Pediatrics and Adolescent Medicine, American University of Beirut, Beirut, Lebanon; 1036 Department of Microbiology, Faculty of Medicine and Allied Sciences, Rajarata University of Sri Lanka, Saliyapura, Sri Lanka; 1037 Research Institute of Influenza, St. Petersburg, Russian Federation; 1038 Queen Sirikit National Institute of Child Health, Bangkok, Thailand; 1039 Department of Microbiology, Faculty of Medicine, Eduardo Mondlane University, Maputo, Mozambique; 1040 Instituto Nacional de Investigación en Salud Publica, Guayaquil, Ecuador; 1041 Universidad Agraria del Ecuador, Guayaquil, Ecuador; 1042 Center of Excellence in Clinical Virology, Faculty of Medicine, Chulalongkorn University, Bangkok, Thailand; 1043 Beijing Key Laboratory of Pediatric Respiratory Infection Diseases, Key Laboratory of Major Diseases in Children, Ministry of Education, National Clinical Research Center for Respiratory Diseases, National Key Discipline of Pediatrics (Capital Medical University), Beijing Pediatric Research Institute, Beijing Children’s Hospital, Capital Medical University, National Center for Children’s Health, Beijing, China; 1044 Ministerio de Salud Pública del Ecuador, Quito, Ecuador; 1045 Department of Virology, Noguchi Memorial Institute for Medical Research, University of Ghana, Legon, Accra, Ghana; 1046 Department of Microbiology, University of Ghana Medical School, Accra, Ghana; 1047 Global Disease Detection Center, Global Disease Detection and Response Program, US Naval Medical Research Unit, US Centers for Disease Control and Prevention, Cairo, Egypt; 1048 Center for Vaccine Development and Global Health, University of Maryland School of Medicine, Baltimore, Maryland, USA; 1049 Faculty of Health Sciences, Universitat Oberta de Catalunya, Barcelona, Spain; 1051 ISGlobal, Hospital Clínic—Universitat de Barcelona, Barcelona, Spain; 1052 Department of Pediatrics and Intensive Care, Fundación Cardioinfantil-Instituto de Cardiología, Universidad de La Sabana, Bogotá, Colombia; 1053 Department of Pediatric Critical Care Medicine, Instituto Roosevelt, Bogotá, Colombia; 1054 Department of Microbiology, Sanjay Gandhi Postgraduate Institute of Medical Sciences, Uttar Pradesh, India; 1055 Department of Microbiology, King George’s Medical University, Lucknow, Uttar Pradesh, India; 1056 University of Health Sciences, Department of Pediatric Pulmonology, Dr Gazi Yaşargil Women’s and Children’s Health, Education and Research Hospital, Diyarbakır, Turkey; 1057 Yemen Field Epidemiology Training Program (Yemen-FETP), Sana’a, Yemen; 1058 Tropical Infectious Diseases Research and Education Centre, Department of Medical Microbiology, Faculty of Medicine, University Malaya, Kuala Lumpur, Malaysia; 1059 Pediatric Infectious Diseases Unit, Santa Casa de Sao Paulo, Sao Paulo, Brazil; 1060 Department of Pediatrics, Santa Casa de Sao Paulo, Sao Paulo, Brazil; 1061 Centro de Pediatria Especializada “CRECER”, Santa Cruz de la Sierra, Bolivia; 1062 Department of Paediatrics, Sibu Hospital, Sibu, Sarawak, Malaysia; 1063 Division of Pediatric Critical Care, Ankara University School of Medicine, Ankara, Turkey; 1064 Institut de Recherche en Sciences de la Sante (IRSS), Bobo-Dioulasso, Burkina Faso; 1065 Infectious Diseases Division, International Centre for Diarrhoeal Disease Research, Bangladesh (icddr,b), Dhaka, Bangladesh; 1066 Department of Medical Virology, University of Pretoria, National Health Laboratory Services, Tshwane Academic Division, Pretoria, South Africa; 1067 Department of Pediatrics, Faculty of Medicine, University of Medicine and Pharmacy, Ho Chi Minh City, Vietnam; 1068 Red Colaborativa Pediátrica de Latinoamericá (LARed Network); 1069 Bronchiolitis and Co-Detection (BACON) study, Red Colaborativa Pediátrica de Latinoamericá (LARed Network); 1070 Hospital de Montevideo, Uruguay, San José, Bogotá, Colombia; 1071 Cuidados Intensivos Pediátricos Especializados (CIPe), Casa de Galicia, Montevideo, Uruguay; 1072 Hospital El Carmen de Maipú, Santiago, Chile; 1073 Escuela de Medicina, Universidad Finis Terrae, Santiago, Chile; 1074 Department of Pediatric Critical Care, Nationwide Children’s Hospital, Columbus, Ohio, USA; 1075 Hospital Susana López de Valencia E.S.E., Popoyán, Colombia; 1076 Hospital Militar Central, Bogotá, Colombia; 1077 Fundación Valle de Lili, Cali, Colombia; 1078 Clínica Infantil Colsubsidio, Bogotá, Colombia; 1079 Hospital Infantil Sabará, Sao Paulo, Brazil; 1080 Hospital Tacuarembó, Tacuarembo, Uruguay; 1081 Pediatric Intensive Care Unit, Médica Uruguaya, Montevideo, Uruguay; 1082 Istanbul Medeniyet University, Goztepe Training and Research Hospital, Pediatric Intensive Care Unit, Istanbul, Turkey; 1083 Child Health Research Foundation (CHRF), Dhaka, Bangladesh; 1084 Division of Pediatric Infectious Diseases, Duke University, Durham, North Carolina, USA; 1085 Clinical Virology Unit, CEMIC—CONICET, Instituto Universitario CEMIC, Buenos Aires, Argentina; 1086 Children’s Hospital No. 1, Ho Chi Minh City, Vietnam; 1087 Pediatric Intensive Care Unit, Children’s Hospital of Tunis, Tunis, Tunisia; 1088 L’Hôpital d’Enfants Béchir-Hamza, Tunis, Tunisia

**Keywords:** community death, lower respiratory tract infection, respiratory syncytial virus

## Abstract

**Background:**

Respiratory syncytial virus (RSV) is a leading cause of pediatric death, with >99% of mortality occurring in low- and lower middle-income countries. At least half of RSV-related deaths are estimated to occur in the community, but clinical characteristics of this group of children remain poorly characterized.

**Methods:**

The RSV Global Online Mortality Database (RSV GOLD), a global registry of under-5 children who have died with RSV-related illness, describes clinical characteristics of children dying of RSV through global data sharing. RSV GOLD acts as a collaborative platform for global deaths, including community mortality studies described in this supplement. We aimed to compare the age distribution of infant deaths <6 months occurring in the community with in-hospital.

**Results:**

We studied 829 RSV-related deaths <1 year of age from 38 developing countries, including 166 community deaths from 12 countries. There were 629 deaths that occurred <6 months, of which 156 (25%) occurred in the community. Among infants who died before 6 months of age, median age at death in the community (1.5 months; IQR: 0.8−3.3) was lower than in-hospital (2.4 months; IQR: 1.5−4.0; *P* < .0001). The proportion of neonatal deaths was higher in the community (29%, 46/156) than in-hospital (12%, 57/473, *P* < 0.0001).

**Conclusions:**

We observed that children in the community die at a younger age. We expect that maternal vaccination or immunoprophylaxis against RSV will have a larger impact on RSV-related mortality in the community than in-hospital. This case series of RSV-related community deaths, made possible through global data sharing, allowed us to assess the potential impact of future RSV vaccines.

## Teaser

The global age distribution of RSV-related deaths in the community is unknown. Children dying of RSV in the community are younger than children dying in-hospital in developing countries. We expect the impact of RSV prevention via maternal vaccination or monoclonal antibody prophylaxis on mortality in early life to be higher for community than in-hospital RSV-related deaths.

As part of the global agenda for 2030 set by the United Nations, Sustainable Development Goal (SDG) 3 urgently calls for ending preventable deaths of children under 5 years of age. Globally, respiratory syncytial virus (RSV) is a leading cause of death after malaria for infants [1]. More than 99% of these RSV pediatric deaths occur in the developing world [2]. Current global mortality estimates are almost exclusively based on in-hospital RSV mortality. However, it is likely that a significant proportion of these deaths occur outside the hospital, especially in low-income settings [3]. A recent meta-analysis estimated that out-of-hospital mortality was 2-fold higher than in-hospital mortality in 3 low-income and lower-middle-income countries (L(M)ICs) [3]. Thus, the burden of out-of-hospital RSV deaths appears to be at least as high as in-hospital deaths. Despite the magnitude of the problem, understanding the clinical characteristics of pediatric RSV-related mortality in the community remains a key knowledge gap.

Addressing the knowledge gap on community deaths can give key insights to inform policy for a future RSV vaccine. More than 50 vaccine candidates are in clinical development for RSV [4]. Different approaches to RSV prevention confer varying degrees and duration of protection. Currently, 2 major approaches are in development for infants: (1) maternal vaccination and (2) passive antibody prophylaxis. Recent late-phase trial data show the potential degree and duration of protection for these different approaches, with infant monoclonals giving a higher degree and duration of protection than a maternal vaccine. The recently published phase III results of a post-fusion F protein maternal RSV vaccine show an antibody half-life of 49.1 days with 44.4% efficacy (95% confidence interval [CI]: 19.6-61.5%) against severe RSV lower respiratory tract infection (LRTI) through the first 3 months of life [5]. Prophylaxis with an extended half-life monoclonal antibody shows a longer duration of protection with 70.1% efficacy (95% CI: 52.3–81.2%) against RSV LRTI through the first 5 months of life [6]. To estimate the potential impact of RSV-preventive interventions against mortality in the developing world, it is essential to characterize children dying of RSV in the community.

Studying community deaths is difficult given the challenges associated with virological studies in deaths that occur in the community. To date, the largest case series of community RSV-related deaths includes 11 deaths at home in a single, urban setting in Argentina [7]. Although in-hospital deaths are challenging to capture in L(M)ICs given the lack of diagnostic capacity, capturing community deaths is even more challenging due to difficulty in ascertaining cause-of-death based on the low specificity of verbal autopsy data and difficulty obtaining postmortem patient samples. However, in the past years, several studies supported by the Bill & Melinda Gates Foundation (BMGF) aimed to measure RSV mortality in the community: Z-PRIME (the Zambia Pertussis RSV Infant Mortality Estimation study); community-based studies in Argentina, India, and Pakistan; as well as the Child Health and Mortality Prevention Surveillance (CHAMPS) in South Africa, Bangladesh, Kenya, Mali, Mozambique, Ethiopia, and Sierra Leone [8]. The RSV Global Online Mortality Database (RSV GOLD) provides the unique opportunity to pool data from all of these studies and compare global individual-level patient data of children dying in the community to children dying in-hospital.

Respiratory syncytial virus–preventive interventions aim to prevent infant death in accordance with the SDGs. Estimated impact of a maternal vaccine or infant monoclonal on pediatric deaths will guide policy decisions and accelerate access to life-saving interventions. The primary aim of this article is to describe global community pediatric deaths under 6 months and compare this group with in-hospital deaths in upper-middle-income countries (UMICs) and L(M)ICs.

## METHODS

### Study Site, Design, and Population

RSV GOLD is a global online registry for children under the age of 5 years who died with laboratory-confirmed RSV infection after 1 January 1995 [9]. Individual patient-level data are collected using an online questionnaire. Variables collected in the RSV GOLD database have been published previously [9]. Data are collected through active outreach to researchers and physicians worldwide. Investigators of BMGF-funded community mortality studies were specifically asked to share data collected through 2 March 2021. The data from these community studies have been published in this supplement issue. Two community studies (Z-PRIME and Pakistan Community Mortality studies) included children younger than 6 months of age; other studies recruited children through at least 12 months of age (Supplementary Table 1). Data from studies submitted to the RSV GOLD registry were collected both prospectively and retrospectively.

**Figure 1.**
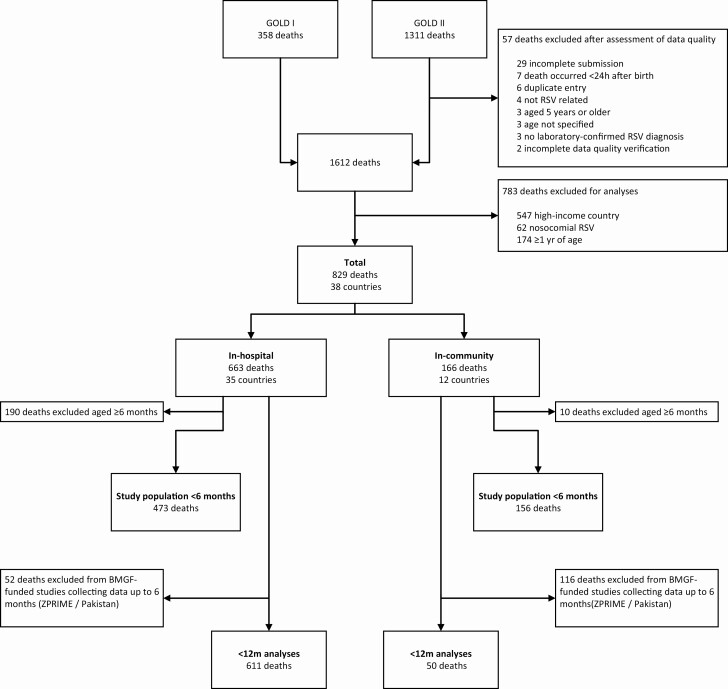
Flowchart of children included in this study. Flowchart shows children excluded via both data quality and per definition of study population. For the primary analysis we analyzed 629 children dying under age 6 months (473 in-hospital deaths and 156 community deaths). For the secondary analysis we analyzed 661 children dying under age 12 months (611 in-hospital deaths and 50 community deaths). GOLD I: Pediatric deaths published as a retrospective case series from 1 November 2014 to 31 October 2015 [9]. GOLD II includes pediatric deaths collected after this publication. Abbreviations: m, months; BMGF, Bill & Melinda Gates Foundation; GOLD, Global Online Mortality Database; RSV, respiratory syncytial virus; ZPRIME, Zambia Pertussis RSV Infant Mortality Estimation Study.

In this analysis RSV-related deaths above 1 year of age, nosocomial deaths, and deaths in high-income countries were excluded (Figure 1). Based on the expected duration of protection for infant RSV-preventive interventions, the primary aim of this study was to compare the age distribution of RSV-related infant deaths under 6 months occurring in the community with those in-hospital. The secondary aim was to describe age at death for children dying of RSV in the first year of life in the community. In order to achieve our secondary aim, to describe the age distribution under 1 year, we analyze the population (“12m cohort”), in which we excluded 2 community studies that only enrolled children up to 6 months of age.

### Data Collection and Case Definition

Case definitions of a community death varied between different BMGF-funded community mortality studies (Supplementary Table 1). For community deaths submitted to RSV GOLD that did not originate from these studies, a community death was defined as a child who did not die in the hospital or a child who was not hospitalized and location of death was unknown (n = 2). As in our previous publications, we included any death with laboratory-confirmed RSV infection and did not require RSV to be the primary cause of death (Supplementary Table 5) [9]. Neonates were defined as children through 1 month of age.

Upon submission to the database, data-quality checks were performed by the RSV GOLD team to ensure the completeness and accuracy of the data. To this end, case data were verified for missingness, plausibility, and accuracy through direct communication with collaborators as soon as possible after case submission. Minimum essential data for inclusion were the key variables age at death, year of death, and laboratory-confirmed RSV infection.

### Statistical Analyses

For continuous variables, the means or medians were reported and differences between 2 groups were tested with a Mann-Whitney *U* test. Categorical variables were described with frequencies and percentages and compared between groups using Fisher’s exact test. We did not perform imputation for missing data because data were not missing for essential variables and for other variables there was no clear correlation on which to build a multiple imputation model.

We considered *P* < .05 to be significant for all analyses. Despite multiple comparisons, we chose not to correct for an increased false-positive rate due to the exploratory nature of the study and small sample size. The statistical analysis was performed using R version 4.0.2 (R Core Team 2020, Vienna, Austria) with the following packages: ggplot2 [10], ggpubr [11], rnaturalearthdata [12], dplyr [13], and qwraps2 [14].

We performed 2 sensitivity analyses: (1) without the Z-PRIME data and (2) restricting the data to community mortality studies. As the majority of the data for community deaths originated from the Z-PRIME study in Zambia, we performed a sensitivity analysis that excluded the Z-PRIME cases to ensure that this overrepresentation of Zambia deaths did not lead to different results. Furthermore, we tested the assumption that community deaths from the Z-PRIME data are representative for community deaths from other L(M)ICs by testing the observed characteristics for significant differences.

### Ethical Considerations

The institutional research board of the University Medical Center Utrecht waived the requirement for parental informed consent in 2014 since the study concerns only anonymized secondary data. Collaborators sharing data were encouraged to adhere to local standards for ethics approval in accordance with the RSV GOLD Ethics Guideline [15].

## RESULTS

### Study Population

**Figure 2.**
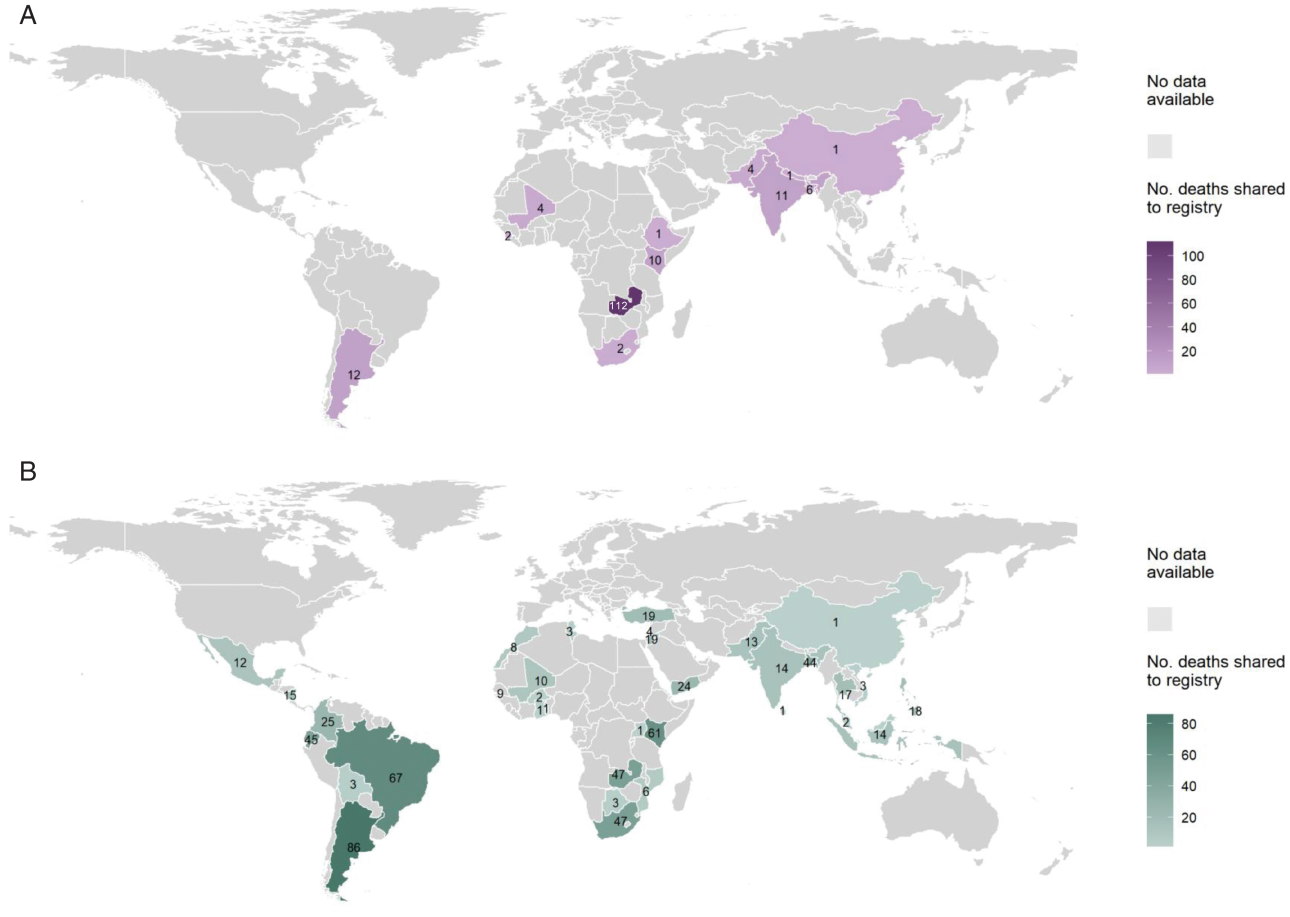
*A*, World map showing L(M)ICs and UMICs that shared RSV-confirmed community deaths under 12 months of age and number of RSV-confirmed community deaths shared to the registry. The color gradient of purple indicates number of deaths shared, with darker purple representing increased number of deaths shared. Numbers of deaths are visible on the map. *B*, World map showing L(M)ICs and UMICs that shared RSV-confirmed in-hospital deaths under 12 months of age and number of deaths of RSV-confirmed in-hospital deaths shared to the registry. The color gradient of green indicates number of deaths shared, with darker green representing increased number of deaths shared. Numbers of deaths are visible on the map. Abbreviations: L(M)ICs, lower-income-lower-middle-income country; RSV, respiratory syncytial virus; UMIC, upper-middle-income country.

The overall study population included 829 pediatric deaths under 1 year of age from 38 countries classified as UMIC or L(M)IC according to the World Bank income group classification (Figure 1, Supplementary Table 2). Of these, 166 deaths occurred in the community. The world maps in Figure 2A and 2B show the global distribution of community and in-hospital deaths, respectively. The study population of infants under 6 months consisted of 629 deaths, of which 156 (25%) deaths from 12 different countries occurred in the community (Supplementary Table 4). Most community deaths were from Zambia (72%, 112/156). Community deaths were submitted from 2009 onwards, while data for in-hospital deaths were shared from 1995 onwards. The 12m cohort comprises 661 children, of whom 8% (50/661) died in the community and 92% (611/661) died in-hospital.

### Age at Death of Community Versus In-Hospital Deaths

**Figure 3.**
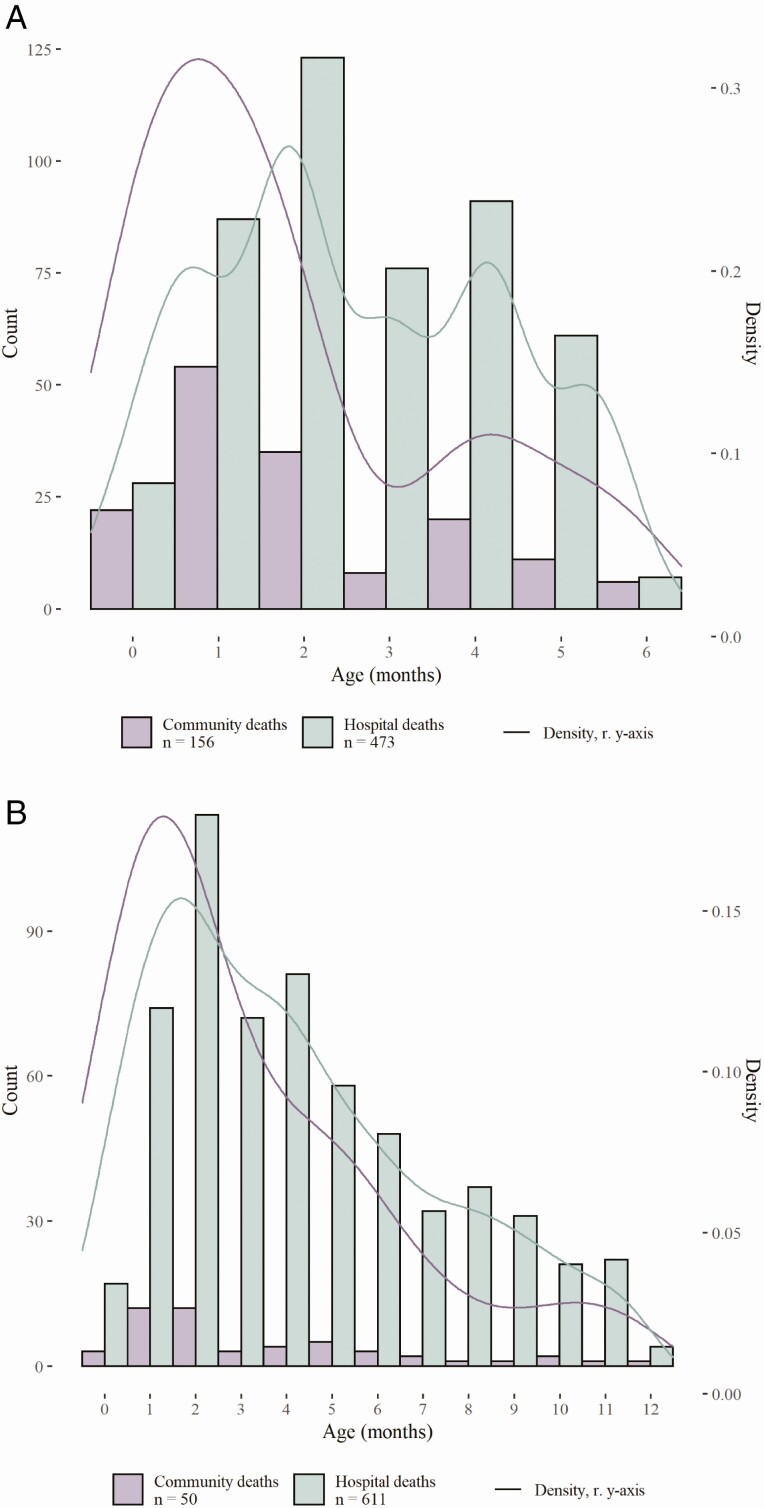
*A*, Histogram and density plot of age at death for children under 6 months who died with RSV in the community compared with in-hospital in L(M)ICs and UMICs. The histogram shows number of deaths (count, left *y*-axis) shared to the registry by age at death in months (rounded to the nearest integer) from age 0 up to 6 months for all infants under 6 months of age. Lines show the kernel density estimate of age at death in months (density, right *y*-axis). Deaths that occurred in the community are shown in purple, while deaths that occurred in the hospital are shown in green. *B*, Histogram and density plot of age at RSV-related death for children under 12 months who died in the community compared with in-hospital in L(M)ICs and UMICs. The histogram shows number of deaths shared (count, left *y*-axis) to the registry by age at death from age 0 up to 12 months for the 12m cohort. Lines show the kernel density estimate of age at death in months (density, right *y*-axis). Deaths that occurred in the community are shown in purple, while deaths that occurred in the hospital are shown in green. Abbreviations: L(M)ICs, lower income and lower middle income country; RSV, respiratory syncytial virus; UMIC, upper-middle-income country.

Median age at death was significantly lower for community deaths (1.5 months; IQR: 0.8−3.3) than in-hospital deaths (2.4 months; IQR: 1.5−4.0; *P* < .0001) (Table 1, Figure 3A). Deaths in the community included a higher proportion of neonates (29%, 46/156) than deaths occurring in-hospital (12%, 57/473; *P* < .0001). Similar results were found for the 12m cohort (Table 2, Figure 3A). In the 12m cohort, median age at death was lower in the community (2.1 months; IQR: 1.3–5.0) compared with in-hospital (4.0 months; IQR: 2.0–6.1; *P* = .02) (Table 2, Figure 3B). Similarly, for the 12m cohort, a higher proportion of deaths occurred in the neonatal period in the community (14%, 7/50) than in-hospital (7%, 40/611), although this difference was not statistically significant (*P* = .08).

**Table 1.** Clinical Characteristics of Children Under 6 Months Who Died with Respiratory Syncytial Virus In-Hospital Versus in the Community in Lower-income Middle-Income Countries and Upper-Middle-Income Countries

Clinical Characteristics	All Deaths (n = 629)	Community (n = 156)	In-Hospital (n = 473)	*P*
Sex, male, % (n/N)	54 (330/615)	55 (78/142)	53 (252/473)	NS
Age at death, months, median (IQR)	2.0 (1.1-4.0)	1.5 (0.8-3.3)	2.4 (1.5-4.0)	<.0001
Neonatal deaths, % (n/N)	16 (103/629)	29 (46/156)	12 (57/473)	<.0001
Comorbidity, % (n/N)	43 (173/403)	31 (11/35)	44 (162/368)	NS
Prematurity, % (n/N)	31 (92/297)	25 (11/44)	32 (81/253)	NS
Gestational age, weeks, mean (SD, n)	36.6 (3.5, 145)	38.5 (2.4, 23)	36.2 (3.6, 122)	.005
Birth weight, kg, median (IQR, n)	2.8 (2.2-3.2, 156)	3.0 (2.4-3.3, 30)	2.8 (2.2-3.2, 126)	NS
Month and year of death, minimum–maximum	July 1995–February 2021	February 2009–July 2020	July 1995–February 2021	…
Not immunized, % (n/N)	30 (71/235)	36 (15/42)	29 (56/193)	NS
Other children in household, % (n/N)	75 (118/158)	82 (18/22)	74 (100/136)	NS
Mother uneducated, % (n/N)	10 (23/231)	6 (6/103)	13 (17/128)	NS
Father uneducated, % (n/N)	6 (9/161)	1 (1/86)	11 (8/75)	.01

*P* values are provided for the comparison between community and in-hospital deaths. Abbreviations: IQR, interquartile range; NS, not significant; SD, standard deviation.

**Table 2.** Clinical Characteristics of Children Under 12 Months Who Died with Respiratory Syncytial Virus In-Hospital Versus in the Community in Lower-Middle-Income Countries and Upper-Middle-Income Countries, Excluding Deaths From Studies Recruiting Only Those Under 6 Months

Clinical Characteristics	All Deaths (n = 661)	Community (n = 50)	In-Hospital (n = 611)	*P*
Sex, male, % (n/N)	56 (369/661)	56 (28/50)	56 (341/611)	NS
Age at death, months, median (IQR)	4.0 (2.0-6.0)	2.1 (1.3-5.0)	4.0 (2.0-6.1)	.02
Neonatal deaths, % (n/N)	7 (47/661)	14 (7/50)	7 (40/611)	NS
Deaths <6 months, % (n/N)	70 (461/661)	80 (40/50)	69 (421/611)	NS
Comorbidity, % (n/N)	45 (250/561)	28 (10/36)	46 (240/525)	.04
Prematurity, % (n/N)	28 (101/356)	24 (9/37)	29 (92/319)	NS
Gestational age, weeks, mean (SD, n)	36.6 (3.5, 195)	38.4 (2.5, 27)	36.3 (3.5, 168)	.01
Birth weight, kg, median (IQR, n)	2.8 (2.2-3.2, 208)	3.0 (2.5-3.3, 30)	2.8 (2.2-3.2, 178)	NS
Month and year of death, minimum–maximum	July 1995–February 2021	February 2009–February 2020	July 1995–February 2021	…
Not immunized, % (n/N)	13 (33/258)	19 (5/27)	12 (28/231)	NS
Other children in household, % (n/N)	73 (160/220)	90 (19/21)	71 (141/199)	NS
Mother uneducated, % (n/N)	12 (19/155)	8 (2/25)	13 (17/130)	NS
Father uneducated, % (n/N)	7 (6/81)	5 (1/21)	8 (5/60)	NS

*P* values are provided for the comparison between community and in-hospital deaths. Abbreviations: IQR, interquartle range; NS, not significant; SD, standard deviation.

### Clinical Characteristics of Community vs In-Hospital Deaths

For infants who died under 6 months, clinical characteristics of community and in-hospital deaths were largely comparable. For children under 6 months with comorbidity data, 31% (11/35) of infants dying in the community had a comorbidity compared with 44% (162/368) who died in-hospital. However, data on comorbidities were missing for 78% of community deaths, limiting the power to analyze this characteristic (comorbidities are specified in Supplementary Table 8). The proportion of premature infants did not differ significantly between community (25%, 11/44) and in-hospital (32%, 81/253; not significant) deaths. We note that prematurity data were missing for a substantial proportion of community (72% 112/156) and in-hospital (47%, 220/473) deaths for infants under 6 months. The reported mean gestational age was lower for deaths in-hospital compared with those occurring in the community (36.2 vs 38.5 weeks; *P* = .005).

The secondary analysis of the 12m cohort was remarkably similar to the primary analysis of children dying before age 6 months. Among infants dying in the community, 28% (10/36) had a comorbidity compared with 46% (240/525, *P* = .04) of infants dying in-hospital. In the 12m cohort, the proportion of premature infants did not differ significantly between community versus in-hospital deaths (Table 2), although reported gestational age was significantly lower for infants dying in-hospital than in the community (36.3 vs 38.4 weeks; *P* = .006).

### Sensitivity Analyses

We performed a sensitivity analysis excluding the majority of the community deaths, which originated from a single Zambian study site (71%, 112/156). The age at death in the community did not differ significantly for children who died in Zambia (n = 112; data shown in Gill et al in this supplement issue) compared with children from other countries (n = 44; 2.0 months; IQR: 1.3–3.3 months). The proportion of children with prematurity was similar for community deaths in Zambia and community deaths elsewhere (Forman et al, data published elsewhere in this supplement issue). After excluding the Zambia data, we found that age at death remained lower for children who died in the community (2.0 months; IQR: 1.3–3.3) compared with children who died in-hospital (2.5 months; IQR: 1.8–4.0), although this difference was not statistically significant (*P* = .07) (Supplementary Table 6). The proportion of neonates was similar in the in-hospital and community deaths (Supplementary Table 6).

We performed a sensitivity analysis restricted to data obtained from the community mortality study sites (144 community deaths and 68 in-hospital deaths) to rule out bias due to differences in methodology of data collection, because data in this subset were collected systematically in the community and in the hospital setting (Supplementary Table 7). In this analysis, we observed a lower median age at death in the community compared with in-hospital, although differences were smaller than in the main analysis and not statistically significant (1.5 vs 2.0 months; *P* = .26). Moreover, in this sensitivity analysis, the proportion of neonates was similar in the in-hospital and community deaths (Supplementary Table 7).

## Discussion

As a result of global data sharing by collaborators, this study is the first global case series to compare RSV-related mortality in the community with in-hospital deaths in L(M)ICs and UMICs. The aim of this study was to understand differences between infants dying in the community and infants dying in-hospital in order to inform RSV vaccine–development strategies for low-resource settings. We found that children dying in the community were generally younger than children dying in-hospital. A larger proportion of deaths in the community involved neonates in the primary analyses but not the sensitivity analyses, possibly due to a larger proportion of deaths originating from L(M)ICs in the community. The younger age at death in the community may be explained by difficulty of caregivers in recognizing respiratory danger signs at a younger age, resulting in delayed or no access to care for younger children with RSV LRTI. We conclude that RSV-prevention strategies targeting infants in the first months of life will likely have a larger impact on mortality occurring in the community than in-hospital. Thus, we expect a high impact of infant RSV immunization strategies via maternal vaccination or infant immunoprophylaxis.

The RSV GOLD database serves as a platform that can bundle data from study sites around the world to allow for a high-level analysis of RSV-related mortality around the globe. Previous publications on community deaths did not describe age distribution for RSV-related illness but instead described risk factors for community deaths in Argentina [7], leading causes of deaths determined by minimally invasive autopsies in the CHAMPS sites [8], and estimates of the proportion of out-of-hospital deaths in South Africa [16]. Previously we published a case series of in-hospital deaths in which we found the median age at death to be 5 months in L(M)ICs and 4 months in UMICs in children under 5 years of age. In this analysis, we found the median age at RSV-related death in infants from UMICs and L(M)ICs who died before 1 year of age to be similar (4.0 months; IQR: 2.0−6.1).

There were several limitations of this study. First, the community mortality studies contributing to the RSV GOLD registry were not designed identically and used different definitions for community deaths (Supplementary Table 1). A second limitation concerns the reporting of age at death by collaborators. There are 2 ways in which collaborators may have rounded age at death to age in months, which could introduce bias in our analysis. Due to general conceptualization of age, collaborators may have rounded age in months down. This rounding method may have introduced systematic bias for the group of children who died in-hospital because age at death was most frequently shared in months for in-hospital deaths and in days for community deaths. Second, collaborators may round age to the nearest integer, which would mean that the cutoffs applied for our analyses exclude children whose age was rounded to 1 (neonates), 6 (primary analysis), or 12 (secondary analysis) months. For example, the observed difference in proportion of neonatal deaths could be influenced by misclassification bias. To this end, age reported in months may have been rounded to age 1 month for deaths in the first month of life and subsequently these children would not be classified as neonatal deaths more often for the in-hospital group. In summary, for both rounding methods, age at death may have been underestimated for children dying in the hospital, which would mean that the difference in age between community and in-hospital deaths may have been underestimated. A third limitation of the study is the quality and completeness of the data. An inherent weakness in global data sharing is that primary data cannot be verified and data-collection systems differ in quality. With extensive data-quality checks and direct verification with collaborators, we attempted to limit the impact of this methodological weakness. For some variables (comorbidity, prematurity), a high proportion of data were missing.

More than 50% of community death data originate from a single study site (Zambia). Our conclusions regarding age at death did not change when excluding these deaths from the analysis. An important limitation is the difficulty of measuring mortality in the community, which may have resulted in missed deaths. Data from the community were from a small number of countries while hospital deaths were shared from a larger number of countries over a longer period of time, which may account for differences in the data. Data from most studies were obtained from systematic postmortem sampling, which may not be comparable to the way in which data were obtained for the in-hospital group and which could explain the different findings in the sensitivity analyses. For this reason, the age difference could also be explained by limitations in study methodology as children with RSV may present with nonspecific symptoms to the hospital at a younger age and not be tested in L(M)ICs. However, in a sensitivity analyses limited to comparable groups in-hospital and in the community, we observed the same trend of lower age at death in the community. Despite data-quality verification processes, there are major limitations of the study methodology as published previously [9].

Future steps should consist of analysis of a larger case series including more community deaths and a larger global distribution, which will allow for more robust conclusions regarding vaccine impact on infant mortality. Prospective, real-time data sharing of RSV-related death in L(M)ICs will contribute to increased data quality and completeness of data, including more detailed information on age at death, allowing for a better comparison between community and in-hospital RSV-related deaths. A uniform definition of RSV-related deaths in the community will allow for better collection and understanding of global community mortality. Future studies would be strengthened by enhanced systems for data collection of key clinical characteristics such as immunization status, prematurity, and comorbidity for community deaths, as this information was frequently missing for this population.

### Conclusions

Community deaths are thought to represent more than half of all RSV-related deaths globally [17]. Characterizing these deaths is essential to estimate the impact of future preventive interventions. Due to global data sharing and efforts of BMGF-funded community mortality studies and other collaborators, the RSV GOLD database has served as a platform to aggregate robust data for analysis of RSV-related pediatric mortality on a global level. We show that infants under 6 months of age die at a younger age in the community than in-hospital. Modeling studies will have to translate these findings into expected impact of upcoming maternal vaccines and next-generation monoclonal antibodies against RSV. For the first time, we show evidence that maternal vaccination or infant monoclonal prophylaxis may have a greater impact on RSV-related community mortality than in-hospital mortality. Ultimately, clinical trials and postmarketing surveillance studies will provide further evidence to evaluate the impact of these interventions on pediatric RSV mortality in the community versus in-hospital.

## Supplementary Data

Supplementary materials are available at *Clinical Infectious Diseases* online. Consisting of data provided by the authors to benefit the reader, the posted materials are not copyedited and are the sole responsibility of the authors, so questions or comments should be addressed to the corresponding author.

ciab528_suppl_Supplementary_Material

## Notes

***Acknowledgments.*** We thank Prachi Vora and Padmini Srikantiah for their scientific advice and support. We thank all members of the RSV GOLD team: Juliette Bollemeijer, Trisja Boom, Amber ten Buuren, Sophie Croon, Dora van Duijvendijk, Nynke van Haastregt, Jasper van der Kemp, Giovanni Loe-Sack-Sioe, Lotte Mesker, Ichelle van Roessel, Dunja Scheepmaker, Femke Vernooij, and Renate Visser.

***Financial support.*** This publication is based on research funded in part by the Bill & Melinda Gates Foundation (grant number OPP1148988.8). ES reports grants from Bill and Melinda Gates Foundation, during the conduct of the study; grants, personal fees and non-financial support from Astra Zeneca Inc, grants, personal fees and non-financial support from Merck & Co., grants, personal fees and non-financial support from Regeneron Inc, grants, personal fees and non-financial support from Pfizer Inc, personal fees, non-financial support and other from Abbvie Inc, personal fees from Alere Inc, grants, personal fees and non-financial support from Roche Inc, other from GSK Inc, grants from Johnson and Johnson, grants and non-financial support from Novavax Inc, outside the submitted work; FP reports grants and personal fees from JANSSEN, grants and personal fees from NOVAVAX, INC, personal fees from BAVARIAN NORDIC A/S, personal fees from PFIZER, personal fees from SANOFI, personal fees from REGENERON, personal fees from MERCK, outside the submitted work.

***Supplement sponsorship.*** This supplement is sponsored by the Bill and Melinda Gates Foundation.

***Potential conflicts of interest.*** L. J. B. has regular interaction with pharmaceutical and other industrial partners. He has not received personal fees or other personal benefits. The University Medical Centre Utrecht (UMCU) has received major funding (>€100 000 per industrial partner) for investigator-initiated studies from AbbVie, MedImmune, Janssen, the Bill & Melinda Gates Foundation, Nutricia (Danone), and MeMed Diagnostics. UMCU has received major cash or in-kind funding as part of the public-private partnership IMI-funded RESCEU project from GSK, Novavax, Janssen, AstraZeneca, Pfizer, and Sanofi. UMCU has received major funding by GlaxoSmithKline and Julius Clinical for participating in the INFORM study sponsored by MedImmune. UMCU has received minor funding for participation in trials by Regeneron and Janssen from 2015 to 2017 (total annual estimate <€20 000). UMCU received minor funding for consultation and invited lectures by AbbVie, MedImmune, Ablynx, Bavaria Nordic, MabXience, Novavax, Pfizer, and Janssen (total annual estimate <€20 000). L. J. B. is the founding chairman of the ReSViNET Foundation. N. I. M. has regular interaction with pharmaceutical and other industrial partners. She has not received personal fees or other personal benefits. Cheryl Cohen (C. C. has received minor funding from Sanofi and support to attend meetings from Parexel. Sandra Chaves (S. S. C.) has left the Centers for Disease Control and Prevention and is currently working for Sanofi Pasteur, France. All other authors report no potential conflicts. All authors have submitted the ICMJE Form for Disclosure of Potential Conflicts of Interest. Conflicts that the editors consider relevant to the content of the manuscript have been disclosed.

***RSV GOLD collaborators.*** Somsak Thamthitiwat, MD,^1^ Angela Gentile, MD,^2^ Maria Florencia Lucion, MD,^2^ Márcia Rosane Pires, RN,^3^ Fernanda de-Paris, PhD,^3^ Aubree Gordon, PhD,^4^ José Félix Sánchez, MD,^5^ Marilla G. Lucero, PhD,^6^ Socorro P. Lupisan, MD,^6^ Bradford D. Gessner, MD, MPH,^7^ Haoua Tall, MPH,^7^ Natasha Halasa, MD,^8^ Najwa Khuri-Bulos, MD,^9^ D. James Nokes, PhD,^10^ Patrick K. Munywoki, PhD,^10^ Grieven P. Otieno, BSc,^10^ Katherine L. O’Brien, MD,^11^ on behalf of the PERCH Study Group; Hitoshi Oshitani, MD,^12^ Maria Tereza da Costa Oliveira, PhD,^13^ Carla Cecília de Freitas Lázaro Emediato, ^13^ Asad Ali, MBBS, MPH,^14^ Uzma Bashir Aamir, PhD,^15^ Daniel E. Noyola, MD, PhD,^16^ Cheryl Cohen, PhD,^17^ Jocelyn Moyes, MD,^17^ Heloisa Ihle Garcia Giamberardino, MD, Msc,^18^ Jane Melissa Webler,^18^ Patricia Gomes de Matos Bezerra, MD, PhD,^19^ Maria do Carmo Menezes Bezerra Duarte, MD, PhD,^19^ Helen Y. Chu, MD, MPH,^20^ Rashmi Ranjan Das, MD, FCCP,^21^ Martin W. Weber, MD, PhD,^22,23^ Nusrat Homaira, MBBS, MPH, PhD,^24,25^ Adam Jaffe, MBBS, MD, FRCPCH, FRCP, FRACP, FThorSoc,^24^ Katharine M. Sturm-Ramirez, PhD,^25,26^ Wei Su, MD,^27^ Chiang Chun Yuan, MD,^27^ Sandra Chaves, MD, MSc,^28^ Gideon O. Emukule, PhD,^28^ Sergio de Andrade Nishioka, MD, PhD,^29,30^ on behalf of the Influenza Surveillance Team at the Brazil Ministry of Health; Felipe Cotrim de Carvalho, ^29^ Şule Gökçe, MD,^31^ Sonia M. Raboni, MD, PhD,^32^ Michael Hawkes, MD, PhD,^33^ Melina Messaoudi,^34^ Juliet Bryant,^34^ Ghassan S. Dbaibo, MD,^35^ Rima Hanna-Wakim, MD,^35^ J. A. A. Sampath Jayaweera, MBBS, MSc, Mphil, MD, FRSPH,^36^ Kirill Stolyarov, ^37^ Piyarat Suntarattiwong, MD, MPH,^38^ Tufária Mussá, ^39^ Alfredo Bruno, MD, MSc,^40,41^ Domenica de Mora, MSc,^40^ Nasamon Wanlapakorn, MD, PhD,^42^ Zheng de Xie, MD, PhD,^43^ Junhong Ai,^43^ Jenny Ojeda, MSc,^44^ Lida Zamora, MSc,^44^ Evangeline Obodai, PhD,^45,46^ John Kofi Odoom, PhD,^45^ Maha Talaat Ismail,^47^ Andrea Buchwald, MD,^48^ Cristina O’Callaghan-Gordo, BSc,^49,50,51^ Jaime Fernandez-Sarmiento,^52^ Evelyn Obando-Belalcazar,^53^ Tapan Dhole,^54^ Sheetal Verma, ^55^ Aykut Eşki, MD,^56^ G. Ozturk Kartal,^56^ Mohammed Al Amad, MPH,^57^ Abdul Wahed Al Serouri, PhD,^57^ Yoke FunChan, PhD,^58^ Jamal I-Ching Sam, MRCPath,^58^ Daniel Jarovsky, MD,^59^ Daniella Gregória Bomfim Prado da Silva, MD,^60^ José Gareca Perales, ^61^ Teck-Hock Toh, MD,^62^ Jeffrey Lee Soon Yit, MD,^62^ Tanil Kendirli, MD, PhD,^63^ Emrah Gun,^63^ Tani Sagna, PhD,^64^ Serge Diagbouga, PhD, DVM,^64^ Fahmida Chowdhury,^65^ Md Ariful Islam,^65^ Marietjie Venter, MSc, PhD,^66^ Adele Visser,^66^ Minh-Hong Pham,^67^ Pablo Vásquez-Hoyos, MD, MSc,^68,69,70^ Sebastián González-Dambrauskas, MD,^68,69,71^ Franco Díaz Rubio, MD, MBA,^68,72,73^ Todd Karsies, MD, MPH,^69,74^ Eliana Zemanate,^68,69,75^ Ledys Izquierdo,^68,69,76^ Rubén Lasso Palomino, ^68,69,77^ Rosalba Pardo-Carrero, ^68,78^ Reginna Grigolli-Cesar,^68,79^ Soledad Menta,^68,80^ Nicolás Monteverde, MD,^68,81^ Muhterem Duyu, MD,^82^ Senjuti Saha, PhD,^83^ Samir K. Saha, PhD,^83^ Matthew Kelly, MD, MPH,^84^ Marcela Echavarria, ^85^ Tuan Tran,^86^ Aida Borgi,^87^ Ahmed Ayari^88^

1Division of Global Health Protection, Thailand Ministry of Public Health—US Centers for Disease Control and Prevention Collaboration, Nonthaburi, Thailand; 2Department of Epidemiology, Ricardo Gutiérrez Children Hospital, Buenos Aires, Argentina; 3Hospital de Clínicas de Porto Alegre, Universidade Federal do Rio Grande do Sul, Porto Alegre, Brazil; 4Department of Epidemiology, School of Public Health, University of Michigan, Michigan, USA; 5Department of Medicine, Hospital de Referencia Manuel de Jesús, Managua, Nicaragua; 6Research Institute for Tropical Medicine, Muntinlupa City, Philippines; 7Agence de Médecine Préventive, Paris, France; 8Vanderbilt University Medical Center, Nashville, Tennessee, USA; 9Department of Pediatrics, University of Jordan, Aljubeiha, Amman, Jordan; 10Kenya Medical Research Institute, Wellcome Trust Research Programme, Centre for Geographic Medicine Research—Coast, Kilifi, Kenya; 11International Vaccine Access Center, Pneumonia Etiology Research for Child Health (PERCH), Johns Hopkins Bloomberg School of Public Health, Baltimore, Maryland, USA; 12Department of Virology, Tohoku University Graduate School of Medicine, Aoba-ku, Sendai, Miyagi, Japan; 13Health Secretariat of the City of Belo Horizonte, Belo Horizonte, Brazil; 14Department of Pediatrics and Child Health, Aga Khan University, Karachi, Pakistan; 15Department of Virology, National Institute of Health, Islamabad, Pakistan; 16Faculty of Medicine, Department of Microbiology, Universidad Autónoma de San Luis Potosí, San Luis Potosí, Mexico; 17Centre for Respiratory Disease and Meningitis, National Institute for Communicable Diseases, Johannesburg, South Africa; 18Hospital Pequeno Príncipe, Curitiba, Brazil; 19Instituto de Medicina Integral Prof. Fernando Figueira (IMIP), Recife, Brazil; 20Division of Allergy & Infectious Diseases, Department of Medicine, University of Washington, Seattle, Washington, USA; 21All India Institute of Medical Sciences (AIIMS), Bhubaneswar, India; 22Department of Child and Adolescent Health and Development, World Health Organization, Geneva, Switzerland; 23Medical Research Council Laboratories, Fajara, The Gambia; 24School of Women’s and Children’s Health, Faculty of Medicine, University of New South Wales, Sydney, New South Wales, Australia; 25International Centre for Diarrheal Disease Research, Bangladesh (icddr,b), Dhaka, Bangladesh; 26Centers for Disease Control and Prevention, Atlanta, Georgia, USA; 27Can Am International Medical Center, Guangzhou, China; 28National Center for Immunization and Respiratory Diseases, Centers for Disease Control and Prevention, Nairobi, Kenya; 29Department of Transmissible Diseases, Ministry of Health, Brasília, Brazil; 30Instituto Oswaldo Cruz-Fiocruz, Rio de Janeiro, Brazil; 31Department of Pediatrics, General Pediatrics Unit, Ege University, Izmir, Turkey; 32Virology Laboratory, Department of Infectious Diseases, Hospital de Clínicas, Federal University of Paraná, Curitiba, Brazil; 33Department of Pediatrics, Stollery Children’s Hospital, University of Alberta, Edmonton, Alberta, Canada; 34Emerging Pathogens Laboratory—Fondation Mérieux, Centre International de Recherche en Infectiologie, Lyon, France; 35Department of Pediatrics and Adolescent Medicine, American University of Beirut, Beirut, Lebanon; 36Department of Microbiology, Faculty of Medicine and Allied Sciences, Rajarata University of Sri Lanka, Saliyapura, Sri Lanka; 37Research Institute of Influenza, St. Petersburg, Russian Federation; 38Queen Sirikit National Institute of Child Health, Bangkok, Thailand; 39Department of Microbiology, Faculty of Medicine, Eduardo Mondlane University, Maputo, Mozambique; 40Instituto Nacional de Investigación en Salud Publica, Guayaquil, Ecuador; 41Universidad Agraria del Ecuador, Guayaquil, Ecuador; 42Center of Excellence in Clinical Virology, Faculty of Medicine, Chulalongkorn University, Bangkok, Thailand; 43Beijing Key Laboratory of Pediatric Respiratory Infection Diseases, Key Laboratory of Major Diseases in Children, Ministry of Education, National Clinical Research Center for Respiratory Diseases, National Key Discipline of Pediatrics (Capital Medical University), Beijing Pediatric Research Institute, Beijing Children’s Hospital, Capital Medical University, National Center for Children’s Health, Beijing, China; 44Ministerio de Salud Pública del Ecuador, Quito, Ecuador; 45Department of Virology, Noguchi Memorial Institute for Medical Research, University of Ghana, Legon, Accra, Ghana; 46Department of Microbiology, University of Ghana Medical School, Accra, Ghana; 47Global Disease Detection Center, Global Disease Detection and Response Program, US Naval Medical Research Unit, US Centers for Disease Control and Prevention, Cairo, Egypt; 48Center for Vaccine Development and Global Health, University of Maryland School of Medicine, Baltimore, Maryland, USA; 49Faculty of Health Sciences, Universitat Oberta de Catalunya, Barcelona, Spain; 50Centro de Investigação em Saúde de Manhiça (CISM), Maputo, Mozambique; 51ISGlobal, Hospital Clínic—Universitat de Barcelona, Barcelona, Spain; 52Department of Pediatrics and Intensive Care, Fundación Cardioinfantil-Instituto de Cardiología, Universidad de La Sabana, Bogotá, Colombia; 53Department of Pediatric Critical Care Medicine, Instituto Roosevelt, Bogotá, Colombia; 54Department of Microbiology, Sanjay Gandhi Postgraduate Institute of Medical Sciences, Uttar Pradesh, India; 55Department of Microbiology, King George’s Medical University, Lucknow, Uttar Pradesh, India; 56University of Health Sciences, Department of Pediatric Pulmonology, Dr Gazi Yaşargil Women’s and Children’s Health, Education and Research Hospital, Diyarbakır, Turkey; 57Yemen Field Epidemiology Training Program (Yemen-FETP), Sana’a, Yemen; 58Tropical Infectious Diseases Research and Education Centre, Department of Medical Microbiology, Faculty of Medicine, University Malaya, Kuala Lumpur, Malaysia; 59Pediatric Infectious Diseases Unit, Santa Casa de Sao Paulo, Sao Paulo, Brazil; 60Department of Pediatrics, Santa Casa de Sao Paulo, Sao Paulo, Brazil; 61Centro de Pediatria Especializada “CRECER”, Santa Cruz de la Sierra, Bolivia; 62Department of Paediatrics, Sibu Hospital, Sibu, Sarawak, Malaysia; 63Division of Pediatric Critical Care, Ankara University School of Medicine, Ankara, Turkey; 64Institut de Recherche en Sciences de la Sante (IRSS), Bobo-Dioulasso, Burkina Faso; 65Infectious Diseases Division, International Centre for Diarrhoeal Disease Research, Bangladesh (icddr,b), Dhaka, Bangladesh; 66Department of Medical Virology, University of Pretoria, National Health Laboratory Services, Tshwane Academic Division, Pretoria, South Africa; 67Department of Pediatrics, Faculty of Medicine, University of Medicine and Pharmacy, Ho Chi Minh City, Vietnam; 68Red Colaborativa Pediátrica de Latinoamericá (LARed Network); Bronchiolitis and Co-Detection (BACON) study, Red Colaborativa Pediátrica de Latinoamericá (LARed Network); 70Hospital de Montevideo, Uruguay, San José, Bogotá, Colombia; 71Cuidados Intensivos Pediátricos Especializados (CIPe), Casa de Galicia, Montevideo, Uruguay; 72Hospital El Carmen de Maipú, Santiago, Chile; 73Escuela de Medicina, Universidad Finis Terrae, Santiago, Chile; 74Department of Pediatric Critical Care, Nationwide Children’s Hospital, Columbus, Ohio, USA; 75Hospital Susana López de Valencia E.S.E., Popoyán, Colombia; 76Hospital Militar Central, Bogotá, Colombia; 77Fundación Valle de Lili, Cali, Colombia; 78Clínica Infantil Colsubsidio, Bogotá, Colombia; 79Hospital Infantil Sabará, Sao Paulo, Brazil; 80Hospital Tacuarembó, Tacuarembo, Uruguay; 81Pediatric Intensive Care Unit, Médica Uruguaya, Montevideo, Uruguay; 82Istanbul Medeniyet University, Goztepe Training and Research Hospital, Pediatric Intensive Care Unit, Istanbul, Turkey; 83Child Health Research Foundation (CHRF), Dhaka, Bangladesh; 84Division of Pediatric Infectious Diseases, Duke University, Durham, North Carolina, USA; 85Clinical Virology Unit, CEMIC—CONICET, Instituto Universitario CEMIC, Buenos Aires, Argentina; 86Children’s Hospital No. 1, Ho Chi Minh City, Vietnam; 87Pediatric Intensive Care Unit, Children’s Hospital of Tunis, Tunis, Tunisia; and 88L’Hôpital d’Enfants Béchir-Hamza, Tunis, Tunisia
