# Supplementary material for: Global Respiratory Syncytial Virus–Related Infant Community Deaths
Source: Clin Infect Dis. 2021 Sep 2;73(Suppl 3):S229–37. doi: 10.1093/cid/ciab528 (PMC8411255; doi:10.1093/cid/ciab528)
Supplement: ciab528_suppl_Supplementary_Material [file ciab528_suppl_supplementary_material.docx]

Supplementary Materials

Index of Supplementary Materials:

Table S1. Inclusion and exclusion criteria for community deaths from BMGF-funded community mortality studies

Table S2. Clinical characteristics of all children <12 months who died with RSV in-hospital vs in-community in LMICs and UMICs

Table S3. Countries of origin for RSV-related in-hospital deaths

Table S4. Countries of origin for RSV-related community deaths

Table S5. RSV Diagnostic test used in infants <6 months and the 12m cohort for RSV-related community and in-hospital deaths

Table S6. Sensitivity test without Zambia data of clinical characteristics for all children <6 months who died in-hospital vs in-community in LMICs and UMICs

Table S7. Sensitivity analysis restricted to children from BMGF-funded community mortality studies <6 months who died in-hospital vs in-community in LMICs and UMICs

Table S8. Comorbidities for all children <12 months in whom comorbidities were reported who died with RSV in-hospital and in-community in LMICs and UMICs

Table S1. Inclusion and exclusion criteria for community deaths from BMGF-funded community mortality studies.

| **RSV community mortality study** | **Inclusion criteria** | **Exclusion criteria** | **Definition of community death** |
| --- | --- | --- | --- |
| Z-PRIME |          Age ≥4 days - <6 months at the time of death           Enrollment <48 hours after death |          Age <4 days           Deaths that occurred >48 hours prior to study screening | Death <48 hours of hospitalization |
| CHAMPS |      (Still)births until 60 months at the time of death       Resident of catchment area |          No parental consent           Death or stillbirth reported to the CHAMPS surveillance team >36 hours after death           Legal regulations preclude MITS procedures and CHAMPS data collection           Body buried/cremated/embalmed | Death outside of a health facility or death on arrival |
| Pakistan |          Age between 0-6 months at the time of death including stillbirths |          No parental consent | Died at home or with a traditional birth attendant or at a local health clinic |
| Argentina |          Children <5 years dying at home (outside any health facility) in the catchment area           Certified as “dead on arrival” by a physician at a hospital           Classified as unusual, suspicious or unknown cause and full necropsy was requested by a local prosecutor           Parental consent |          (Suspicion) of criminal cause of death | Death <12 hours of hospitalization or dead on arrival |
| India |          Age less than 2 years           Living in the study area           Informed consent can be obtained from parent or caretaker |          Planning to leave study area within 3 months of time of enrollment           Migration to another village | Death <24 hours of hospitalization |

| **Table S2: Clinical characteristics for all children <12 months who died with RSV in-hospital vs in-community in LMICs and UMICs** | | | |
| --- | --- | --- | --- |
| **Clinical characteristics** | All deaths (n=829) | Community (n=166) | In-hospital (n=663) |
| **Sex, male; % (n/N)** | 54 (443/815) | 54 (82/152) | 54 (361/663) |
| **Age at death, months; median (IQR)** | 3.0 (1.7 – 5.7) | 1.7 (0.9-3.8) | 4.0 (2.0 – 6.0) |
| **Neonatal deaths; % (n/N)** | 12 (103/829) | 28 (46/166) | 9 (57/663) |
| **Deaths <6m; % (n/N)** | 76 (629/829) | 94 (156/166) | 71 (473/663) |
| **Comorbidity; % (n/N)** | 46 (267/583) | 35 (15/43) | 47 (252/540) |
| **Prematurity; % (n/N)** | 30 (121/402) | 27 (14/51) | 30 (107/351) |
| **Gestational age, weeks; mean (SD, N)** | 36.6 (3.5, 197) | 38.4 (2.5, 27) | 36.3 (3.5, 170) |
| **Birth weight, kilograms; median (IQR, N)** | 2.8 (2.2 – 3.2, 217) | 3.0 (2.5 – 3.2, 37) | 2.8 (2.2 – 3.2, 180) |
| **Month & year of death; min/max** | Jul 1995 – Feb 2021 | Feb 2009 – Jul 2020 | Jul 1995 – Feb 2021 |
| **Not immunized; % (n/N)** | 23 (73/315) | 33 (16/48) | 21 (57/267) |
| **Other children in household; % (n/N)** | 74 (173/235) | 85 (22/26) | 72 (151/209) |
| **Mother uneducated; % (n/N)** | 12 (32/273) | 7 (7/107) | 15 (25/166) |
| **Father uneducated; % (n/N)** | 5 (10/184) | 1 (1/90) | 10 (9/94) |

**Table S3. Countries of origin for RSV-related in-hospital deaths**

| Country | BMGF-funded community mortality study | Deaths shared to registry |
| --- | --- | --- |
| Argentina | no | 86 |
| Bangladesh | no | 44 |
| Bolivia | no | 3 |
| Botswana | no | 3 |
| Brazil | no | 67 |
| Burkina Faso | no | 2 |
| China | no | 1 |
| Colombia | no | 25 |
| Ecuador | no | 45 |
| Egypt | no | 18 |
| Gambia | no | 9 |
| Ghana | no | 1 |
| India | no | 12 |
| India | yes | 2 |
| Indonesia | no | 14 |
| Jordan | no | 19 |
| Kenya | no | 60 |
| Kenya | yes | 1 |
| Lebanon | no | 4 |
| Malaysia | no | 2 |
| Mali | no | 6 |
| Mali | yes | 4 |
| Mexico | no | 12 |
| Morocco | no | 8 |
| Mozambique | no | 6 |
| Nicaragua | no | 15 |
| Pakistan | no | 3 |
| Pakistan | yes | 10 |
| Philippines | no | 18 |
| South Africa | no | 33 |
| South Africa | yes | 14 |
| Sri Lanka | no | 1 |
| Thailand | no | 17 |
| Togo | no | 1 |
| Tunisia | no | 3 |
| Turkey | no | 19 |
| Uganda | no | 1 |
| Vietnam | no | 3 |
| Yemen | no | 24 |
| Zambia | no | 5 |
| Zambia | yes | 42 |

Countries shown in the table are location of death. Abbreviations: RSV: respiratory syncytial virus; BMGF: Bill & Melinda Gates Foundation.

| **Table S4. Countries of origin for RSV-related community deaths** | | |
| --- | --- | --- |
| **Country** | BMGF-funded community mortality study | Deaths shared to registry |
| Argentina | yes | 12 |
| Bangladesh | no | 6 |
| China | no | 1 |
| Ethiopia | yes | 1 |
| India | yes | 11 |
| Kenya | no | 5 |
| Kenya | yes | 5 |
| Mali | no | 1 |
| Mali | yes | 3 |
| Nepal | no | 1 |
| Pakistan | yes | 4 |
| Sierra Leone | yes | 2 |
| South Africa | yes | 2 |
| Zambia | yes | 112 |

Countries shown in the table are location of death. Abbreviations: RSV: respiratory syncytial virus; BMGF: Bill & Melinda Gates Foundation.

| **Table S5. RSV diagnostic test used in infants <6 months and the 12m cohort for RSV-related community and in-hospital deaths** | | | |
| --- | --- | --- | --- |
| **Infants <6 months** | All deaths (n=629) | Community (n=156) | In-hospital (n=473) |
| **Diagnostic test used** |  |  |  |
| **Culture** | 25 | 0 | 25 |
| **IF** | 171 | 1 | 170 |
| **IMA** | 19 | 1 | 18 |
| **PCR** | 460 | 156 | 304 |
| **Serology** | 22 | 0 | 22 |
| **Other** | 119 | 1 | 1118 |
| **12m cohort** | All deaths (n=661) | Community (n=53) | In-hospital (n=608) |
| **Culture** | 36 | 0 | 36 |
| **IF** | 251 | 1 | 250 |
| **IMA** | 21 | 1 | 20 |
| **PCR** | 414 | 50 | 364 |
| **Serology** | 29 | 0 | 29 |
| **Other** | 173 | 1 | 172 |

Abbreviations: RSV: respiratory syncytial virus; IF: immunofluorescence; IMA: immunometric assay; PCR: polymerase chain reaction. Of note, one child may have had multiple RSV diagnositic tests reported.

| **Table S6: Sensitivity analysis without Zambia data of clinical characteristics for all children <6 months who died in-hospital vs in-community in LMICs and UMICs** | | | | |
| --- | --- | --- | --- | --- |
| Clinical characteristics | All deaths (n=475) | Community (n=44) | In-hospital (n=431) | p-value |
| Sex, male; % (n/N) | 56 (264/475) | 59 (26/44) | 55 (238/431) | NS |
| Age at deaths, months; median (IQR) | 2.2 (1.6– 4.0) | 2.0 (1.3 – 3.3) | 2.5 (1.8 – 4.0) | 0.07 |
| Neonatal deaths; % (n/N) | 11 (51/475) | 16 (7/44) | 10 (44/431) | NS |
| Comorbidity; % (n/N) | 41 (159/389) | 23 (7/31) | 42 (152/358) | 0.04 |
| Prematurity; % (n/N) | 29 (74/259) | 19 (6/32) | 30 (68/227) | NS |
| Gestational age, weeks; mean(SD, N) | 36.6 (3.5, 145) | 38.5 (2.4, 23) | 36.2 (3.6, 122) | 0.005 |
| Birth weight, kilograms; median (IQR, N) | 2.8 (2.2 – 3.2, 147) | 3.0 (2.6 – 3.3, 23) | 2.8 (2.2 – 3.2, 124) | NS |
| Month & year of death; min/max | Jul 1995 – Feb 2021 | Feb 2009 – Feb 2020 | Jul 1995 – Feb 2021 | – |
| Not immunized; % (n/N) | 23 (44/191) | 32 (8/25) | 22 (36/166) | NS |
| Other children in household; % (n/N) | 75 (118/157) | 86 (18/21) | 74 (100/136 ) | NS |
| Mother uneducated; % (n/N) | 13 (17/127 ) | 8 (2/25) | 15 (15/102) | NS |
| Father uneducated; % (n/N) | 11 (8/72) | 5 (1/21) | 14 (7/51) | NS |

P-value is provided for the comparison between community and in-hospital deaths after excluding all data from the Z-PRIME study. Abbreviations: LMICs: lower-middle income countries; UMICs: upper middle income countries; RSV: respiratory syncytial virus.

| **Table S7: Sensitivity analysis restricted to children from BMGF-funded community mortality studies <6 months who died in-hospital vs in-community in LMICs and UMICs** | | | | |
| --- | --- | --- | --- | --- |
| Clinical characteristics | All deaths (n=212) | Community (n=144) | In-hospital (n=68) | p-value |
| Sex, male; % (n/N) | 51 (101/198) | 55 (71/130) | 44 (30/68) | NS |
| Age at deaths, months; median (IQR) | 1.6 (0.8 – 3.5) | 1.5 (0.8 – 3.3) | 2.0 (0.9 – 3.8) | NS |
| Neonatal deaths; % (n/N) | 30 (63/212) | 31 (44/144) | 28 (19/68) | NS |
| Comorbidity; % (n/N) | 43 (27/63) | 27 (9/33) | 60 (18/30) | 0.01 |
| Prematurity; % (n/N) | 38 (30/79) | 22 (8/37) | 52 (22/42) | 0.006 |
| Gestational age, weeks; mean (SD, N) | 37.2 (3.5, 32) | 38.6 (2.1, 21) | 34.6 (4.2, 11) | 0.005 |
| Birth weight, kilograms; median (IQR, N) | 2.8 (2.4 – 3.2, 43) | 3.0 (2.4 – 3.3, 29) | 2.6 (2.1 – 3.1, 14) | NS |
| Month & year of death; min/max | Sep 2016 – Jul 2020 | Sep 2016 – Jul 2020 | Mar 2017 – Mar 2020 | – |
| Not immunized; % (n/N) | 54 (43/80) | 32 (12/37) | 72 (31/43) | 0.001 |
| Other children in household; % (n/N) | 88 (28/32) | 79 (15/19) | 100 (13/13) | NS |
| Mother uneducated; % (n/N) | 10 (14/139) | 5 (5/101) | 24 (9/38) | 0.003 |
| Father uneducated; % (n/N) | 5 (6/122) | 1 (1/86) | 14 (5/36) | 0.01 |

P-value is provided for the comparison between community and in-hospital deaths restricted to the data obtained via the community mortality study. Abbreviations: LMICs: lower-middle income countries; UMICs: upper middle income countries; RSV: respiratory syncytial virus.

| **Table S8: Comorbidities for all children <12 months who died with RSV in-hospital and in-community in LMICs and UMICs** | | | |
| --- | --- | --- | --- |
| Comorbidity | All deaths (n=583*) | Community (n=43*) | In-hospital (n=540*) |
| Congenital heart disease; n | 97 | 4 | 93 |
| Chronic lung disease; n | 42 | 2 | 40 |
| Immune disorder; n | 12 | 0 | 12 |
| Genetic disease; n | 45 | 4 | 41 |
| Down syndrome; n | 26 | 0 | 26 |
| Neurological disease; n | 42 | 4 | 38 |
| Airway abnormality; n | 3 | 1 | 2 |
| Malaria; n | 8 | 2 | 6 |
| HIV; n | 35 | 4 | 31 |
| Tuberculosis; n | 6 | 0 | 6 |
| Malignancy; n | 2 | 0 | 2 |
| Other comorbidity | 23 | 5 | 18 |
| Other heart disease; n | 2 | 0 | 2 |
| Biliary disease; n | 4 | 0 | 4 |
| Liver disease; n | 2 | 0 | 2 |
| Renal disease; n | 2 | 0 | 2 |
| Congenital abnormality; n | 5 | 0 | 5 |
| Metabolic disorder; n | 1 | 0 | 1 |
| Endocrine disorder; n | 2 | 0 | 2 |
| Sickle cell disease; n | 1 | 1 | 0 |
| Pulmonary hypertension; n | 0 | 0 | 0 |
| Severe malnutrition; n | 5 | 3 | 2 |

Abbreviations: LMICs: lower-middle income countries; UMICs: upper middle income countries; RSV: respiratory syncytial virus, HIV: human immunodeficiency virus. *:Number of deaths is reported in table for children for which comorbidity data was available.
